# Synthesis, Spectroscopic, and Biological Studies on New Zirconium(IV) Porphyrins with Axial Ligand

**DOI:** 10.1155/2013/903616

**Published:** 2013-09-10

**Authors:** Gauri D. Bajju, Gita Devi, Sapna Katoch, Madhulika Bhagat, Deepmala, Ashu, Sujata Kundan, Sunil Kumar Anand

**Affiliations:** ^1^P.G. Department of Chemistry, University of Jammu, Jammu, Jammu and Kashmir 180006, India; ^2^School of Biotechnology, University of Jammu, Jammu, Jammu and Kashmir 180006, India

## Abstract

A series of parasubstituted tetraphenylporphyrin zirconium(IV) salicylate complexes (SA/5-SSAZr(IV)RTPP, R = p-H, p-CH_3_, p-NO_2_, p-Cl, SA = salicylate, and 5-SSA = 5-sulfosalicylate) have been synthesized, and the spectral properties of free base porphyrins, their corresponding metallated, and axially ligated zirconium(IV) porphyrin compounds were compared with each other. A detailed analysis of ultraviolet-visible (UV-vis), proton nulcear magnetic resonance (^1^H NMR) spectroscopy, infrared (IR) spectroscopy, and elemental analysis suggested the transformation from free base porphyrins to zirconium(IV) porphyrins. The ability of the metal in this complex for extra coordination of solvent molecules was confirmed by ESI-MS spectra. Besides the fluorescence, cyclic voltammetry, and thermogravimetric studies, the complexes were also screened for antimicrobial and anticancer activities. Among all the complexes, 5-SSAZr(p-NO_2_TPP) shows high antibacterial activity.

## 1. Introduction

Synthesis and functionalization of porphyrins [1] have long been of great interest in the chemistry community because of the vast potentials and demands for porphyrin derivatives in diverse fields, such as materials [2, 3], supramolecular chemistry [4, 5], biomimetic models [6], catalysis [7, 8], photodynamic therapy [9], and ionophores [10]. Porphyrins were reported to exhibit a variety of biological activities. This is due to the fact that natural and synthetic porphyrins have relatively low toxicity *in vitro* and *in vivo* and they possess antitumor [11, 12] and antioxidant effects [13, 14] and have a good potential for metal ions complexation. The ability for numerous chemical modifications and the large number of different mechanisms by which porphyrins affect microbial and viral pathogens place porphyrins into a group of compounds with an outstanding potential for discovery of novel agents, procedures, and materials active against pathogenic microorganisms [15]. Metalloporphyrins are the basis of new antifungal, antiparasitic, and anticancer drugs because modification of the porphyrin periphery confers qualitatively a new spectrum of activities to metalloporphyrins [16, 17]. It has been reported that metal complexation alters the various physiological properties, especially the cytotoxic and antitumor activities, of many naturally occurring compounds. Zirconium(IV) porphyrins have gained attention from global researchers due to the peculiar characteristics of this class of compounds. To the best of our knowledge, the chemistry of zirconium(IV) porphyrinates remains underdeveloped, being limited to OEP and TPP, with a small variety of derivatives with different anions to balance the remaining +2 charge and ligands to satisfy the 7-8 coordination sphere. The metal ion in these complexes is oxophilic [18]; thus, it may show preference for carboxylate and other oxygen-bearing anionic ligands. A lot of work is reported on the spectral, electrochemical, and biological properties of zirconium and hafnium phthalocyanines with different outplaned organic ligands [19–22], but comparatively less work has been done on zirconium(IV) porphyrin complexes with carboxylate as axial ligand which are expected to be biomedically relevant complexes. Salicylic acid and its derivatives are biologically important molecules, and in view of the interesting results obtained from zirconium(IV) phthalocyanines with various out-of-plane ligands, it is considered worthwhile to make a study of axially substituted zirconium(IV) porphyrin with salicylic acid and its derivatives. We believe that these novel compounds will be fundamental substances for potential applications in the future. With this objective, we aimed at the synthesis, spectroscopic characterization, and biological studies on axially substituted zirconium(IV) porphyrins with salicylic acid and 5-sulfosalicylic acid as axial ligands.

## 2. Experimental

### 2.1. Materials and Instruments

All the chemicals were of analytical grade and used as received unless otherwise noted. Pyrrole was distilled over potassium hydroxide pellets under vacuum prior to use. All the organic solvents that were used for the synthesis and for chromatographic separations were dried before use. (TBA)PF_6_ was recrystallized twice from EtOAc and dried in vacuo prior to use. Elementary analyses (C, H, N, and S) were obtained on a Vario EL III and CHNS-932 Leco Elemental Analyzer. UV-vis spectra were recorded on a T90+ UV/VIS spectrophotometer in the range 350–700 nm. The oscillator strength (*f*) of the transitions in absorption spectra were calculated from the expression

(1) $$\begin{array}{c} f=\;4.33\times 10^{-9}{\varepsilon \Delta \nu }_{1/2}, \end{array}$$

where *ε* is the molar absorption coefficient in dm^3^mol^−1^cm^−1^ and Δ*ν*_1/2_ is the full width at half maximum in cm^−1^. Infrared spectra were recorded on a Perkin Elmer-spectrum 400 FTIR spectrophotometer using KBr pellets in the range of 4000–400 cm^−1^. The ^1^H NMR spectra were recorded on a Bruker Avance II 500 (500 MHz) using tetramethylsilane as internal standard and CDCl_3_ as solvent. TGA and DTA were recorded on Linseis STA PT-100 thermometer using around 21.87 mg of dry samples at the heating rate of 10°C/min in an air atmosphere. The cyclic voltammetry measurements were carried out by an Autolab computer-controlled electrochemical measurement system equipped with a potentiostat. A three-electrode system comprised a gold working electrode, a Pt wire counter electrode, and a saturated Ag/AgCl in KCl as reference electrode. A 0.1 M solution of n-tetrabutylammonium hexafluorophosphate, (TBA)PF_6_ in freshly distilled CH_2_Cl_2_, was used as a supporting electrolyte during the electrochemical experiments. The scan rate was 20 mV/s and the range was −0.2–0.2 mV. The concentration of the porphyrins was 10^−6 ^M. The solutions were purged with oxygen-free nitrogen gas prior to measurements, and all measurements were made at room temperature. Fluorescence measurements were performed on Synergy MX Biotek Multimode Reader. The porphyrins solution prepared in CH_2_Cl_2_ was 10^−6^ M.

### 2.2. Biological Studies 

#### 2.2.1. Antibacterial Studies

Qualitative analysis for screening of antibacterial activity was carried out by Agar well-diffusion method [23] with modifications. The compound was tested against two Gram positive bacteria (*Bacillus subtilis* MTCC2389 and *Staphylococcus aureus* MTCC7443) and three Gram negative bacteria (*Micrococcus luteus* MTCC4821, *Escherichia coli* MTCC2127, and *Pseudomonas fluorescens* MTCC4828). 20 mL of sterilized nutrient agar was inoculated with 100 mL of bacterial suspension (10^8^ CFU/mL) and then poured onto sterilized Petri plate. The agar plate was left to solidify at room temperature. A well of 6 mm was aseptically bored into the agar plate. Then, 20 mL of the complexes (diluted with DMSO, 1 : 1) was added in each well. Chloramphenicol (10 *μ*g) was used as a positive reference to determine the sensitivity of bacteria. The plates were kept at 4°C for 2 hours to allow the dispersal and then incubated at 37°C for 24 hours.

#### 2.2.2. *In Vitro* Cytotoxicity against Human Cancer Cell Lines

*Cell Lines and Cell Cultures.* The human prostrate (PC-3), lung (A-549), and acute lymphoblastic leukemia (THP-1) cell lines were grown and maintained in RPMI-1640 medium, pH 7.4, whereas DMEM was used for breast (MCF-7). The media were supplemented with FCS (10%), penicillin (100 units/mL), streptomycin (100 *μ*g/mL), and glutamine (2 mM), and cells were grown in CO_2_ incubator (Heraeus, GmbH, Germany) at 37°C with 90% humidity and 5% CO_2_. Cells were treated with samples dissolved in DMSO while the untreated control cultures received only the vehicle (DMSO, <0.2%).

*Cytotoxicity Assay. In vitro* cytotoxicity against human cancer cell lines was determined using sulforhodamine B dye assay [24, 25]. Both test samples stock solutions were prepared in DMSO.

### 2.3. Synthesis of Axially Ligated Zirconium(IV) Porphyrins Complexes

#### 2.3.1. Synthesis of Macrocycles

The metal free-base meso-tetraphenylporphyrin (TPP) and its parasubstituted derivative (RTPP, R = p-CH_3_, p-NO_2_, p-Cl) were synthesized by the direct condensation of pyrrole with substituted benzaldehydes according to the documented procedure [26]. The purified porphyrin complexes were obtained in yields of less than 25%.

#### 2.3.2. Synthesis of Dichloro(5,10,15,20-tetraarylporphinato)zirconium(IV), Cl_2_Zr(RTPP)

Dichloro(5,10,15,20-tetraphenylporphinato)zirconium(IV), Cl_2_Zr(RTPP)s were obtained by the reaction of mesotetraphenylporphyrin and its parasubstituted derivatives, RTPP (R = p-H, p-CH_3_, p-NO_2_, p-Cl) with zirconium(IV)chloride by benzonitrile method [27]. RTPP (0.41 mmol) was boiled with ZrCl_4_ (2.64 mmol) in 15 mL of benzonitrile at reflux for 1–3 hours. The reaction course was monitored by absorption spectra of the reaction mixture. The refluxing was stopped when in the electron absorption spectra of the reaction mixture disappeared the absorption bands of RTPP. The complex was isolated in the solid form after distilling benzonitrile off in a vacuum. Then the saturated solution of the complex in chloroform was subjected to chromatography on basic Al_2_O_3_ using CHCl_3_ as eluent. After chromatography, the solution of the zirconium(IV) complex was treated additionally with gaseous HCl. The purified dichlorozirconium(IV) porphyrin complexes were obtained in yields of around 30%.

*(1) Dichloro(5,10,15,20-tetraphenylporphinato)zirconium(IV), Cl*_2_ *ZrTPP.* Red-violet. Anal. Calcd. for C_44_H_28_Cl_2_N_4_Zr(%): C 68.20, H 3.64, N 7.23. Found: C 68.32, H 3.66, N 7.24. UV-vis spectra (CHCl_3_)*λ*_max⁡_: 407 nm (Soret band), 541 nm (Q-band). IR (KBr)*ν*_max⁡_: 485 cm^−1^ (*ν*_Zr−N_). ^1^H NMR (500 MHz, CDCl_3_): 8.87 (8H, s, pyrrole), 8.32 (4H, d, o-phenyl), 8.04 (4H, d, o-phenyl), 7.64 (12H, s, m-phenyl, p-phenyl).

*(2) Dichloro(meso-tetra(p-methylphenyl)porphyrinato)zirconium(IV), Cl*_2_ *Zr(p-CH*_3_ *TPP).* Yellowish brown solid. Anal. Calcd. for C_48_H_36_Cl_2_N_4_Zr(%): C 69.38, H 4.37, N 6.74. Found: C 69.42, H 4.41, N 6.79. UV-vis spectra(CHCl_3_)*λ*_max⁡_: 409 nm (Soret band), 540 nm (Q-band). IR (KBr)*ν*_max⁡_: 508 cm^−1^ (*ν*_Zr−N_). ^1^H NMR (500 MHz, CDCl_3_): 8.80 (8H, s, pyrrole), 8.28 (4H, s, o-phenyl), 8.00 (4H, s, o-phenyl), 7.65 (8H, s, m-phenyl), 2.70 (s, 12H, p-CH_3_).

*(3) Dichloro(meso-tetra(p-chlorophenyl)porphyrinato)zirconium(IV), Cl*_2_ *Zr(p-ClTPP).* Red-violet solid. Anal. Calcd. for C_44_H_24_Cl_6_N_4_Zr(%): C 57.91, H 2.65, N 6.14. Found: C 58.03, H 2.68, N 6.23. UV-Vis spectra (CHCl_3_)*λ*_max⁡_: 416 nm (Soret band), 541 nm (Q-band). IR (KBr)*ν*_max⁡_: 485 cm^−1^ (*ν*_Zr−N_). ^1^H NMR (500 MHz, CDCl_3_): 8.97 (8H, s, pyrrole), 8.33 (4H, d, o-phenyl), 8.13 (4H, s, o-phenyl), 7.59 (8H, s, m-phenyl).

*(4) Dichloro(meso-tetra(p-nitrophenyl)porphyrinato)zirconium(IV), Cl*_2_ *Zr(p-NO*_2_ *TPP).* Reddish brown solid. Anal. Calcd. for C_44_H_24_Cl_2_N_8_O_8_Zr(%): C 55.35, H 2.53, N 11.74. Found: C 55.42, H 2.56, N 11.63. UV-vis spectra (CHCl_3_)*λ*_max⁡_: 414 nm (Soret band), 543 nm (Q-band). IR (KBr)*ν*_max⁡_: 482 cm^−1^ (*ν*_Zr−N_). ^1^H NMR (500 MHz, CDCl_3_): 9.05 (8H, s, pyrrole), 8.42 (4H, s, o-phenyl), 8.04(4H, s, o-phenyl), 7.65(8H, s, m-phenyl).

#### 2.3.3. Synthesis of Axially Ligated Zr(IV) Porphyrins: SAZr(RTPP) and 5-SSAZr(RTPP)

Cl_2_Zr(RTPP) (0.15 mmol) in 25 mL CHCl_3_ was treated with excess of respective salicylate (sodium salicylate/sodium 5-sulfosalicylate) (0.56 mmol) in 25 mL CH_3_OH and stirred under reflux for 12 hours [28]. After concentration, the mixture was dissolved in minimum quantity of CH_2_Cl_2_ and extracted four times with distilled water to remove excess salicylic acid/5-sulfosalicylic acid. The lower layer containing compound in CH_2_Cl_2_ was collected, and then it was filtered through anhydrous Na_2_SO_4_ in order to remove water. The compound was recrystallized from dichloromethane-hexane solution (1 : 1). The same procedure was applied for the synthesis of all axially ligated zirconium porphyrin complexes as described above. The purified axially ligated zirconium porphyrin complexes were obtained in yields of 40–45%.

*(1) SAZrTPP.* Red solid. Anal. Calcd. for C_51_H_32_N_4_O_3_Zr(%): C 72.92, H 3.84, N 6.67. Found: C 72.92, H 3.76, N 6.63. UV-vis (CHCl_3_)*λ*_max⁡_: 418 nm (Soret band), 549 nm, 590 nm (Q-band). ESI-MS (CH_3_OH:CH_3_CN): *m/z *calcd. for C_51_H_32_N_4_O_3_Zr: 840. Found 841 ([M+H]^+^); IR (KBr)*ν*_max⁡_: 485 cm^−1^ (*ν*_Zr−N_), 662 cm^−1^ (*ν*_Zr−O_, phenolic SA), 719 cm^−1^ (*ν*_Zr−O_, carboxylic SA). ^1^H NMR (500 MHz, CDCl_3_): *δ* (ppm) 8.93 (8H, s, pyrrole), 8.50 (4H, s, o-phenyl), 8.07 (4H, d, o-phenyl), 7.68 (12H, m, m-phenyl, p-phenyl), 6.98 (1H, q, 4-phenyl SA), 6.18 (4H, q, 5-phenyl SA), 6.16 (4H, d, 3-phenyl SA), 6.06 (1H, d, 6-phenyl SA).

*(2) 5-SSAZrTPP.* Dark reddish brown solid. Anal. Calcd. for C_51_H_32_N_4_O_6_SZr(%): C 66.57, H 3.51, N 6.09, S 3.48. Found: C 66.53, H 3.64, N 6.07, S 3.48; UV-vis (CHCl_3_)*λ*_max⁡_: 420 nm (Soret band), 549 nm, 587 nm (Q-band). ESI-MS (CH_3_OH:CH_3_CN): *m/z *calcd. for C_51_H_32_N_4_O_6_SZr: 920; found 921 ([M+H]^+^). IR (KBr)*ν*_max⁡_: 500 cm^−1^ (*ν*_Zr−N_), 678 cm^−1^ (*ν*_Zr−O_, phenolic 5-SSA), 722 cm^−1^ (*ν*_Zr−O_, carboxylic 5-SSA). ^1^H NMR (500 MHz, CDCl_3_): *δ* (ppm) 8.96 (8H, s, pyrrole), 8.53 (4H, s, o-phenyl), 8.11 (4H, d, o-phenyl), 7.71 (12H, m, m-phenyl, p-phenyl), 7.10 (1H, q, 4-phenyl 5-SSA), 7.15 (1H, d, 3-phenyl 5-SSA), 7.19 (1H, s, 6-phenyl 5-SSA).

*(3) SAZr(p-CH*_3_ *TPP).* Brown solid. Anal. Calcd. for C_55_H_40_N_4_O_3_Zr(%): C 73.71, H 4.50, N 6.25. Found: C 73.63, H 4.49, N 6.25; UV-vis (CHCl_3_)*λ*_max⁡_: 422 nm (Soret band), 547 nm, 591 nm (Q-band). ESI-MS (CH_3_OH:CH_3_CN): *m/z *calcd. for C_55_H_40_N_4_O_3_Zr: 896. Found 897 ([M+H]^+^). IR (KBr)*ν*_max⁡_: 510 cm^−1^ (*ν*_Zr−N_), 683 cm^−1^ (*ν*_Zr−O_, phenolic SA), 713 cm^−1^ (*ν*_Zr−O_, carboxylic SA). ^1^H NMR (500 MHz, CDCl_3_): *δ* (ppm) 8.94 (8H, s, pyrrole), 8.42 (4H, s, o-phenyl), 8.15 (4H, d, o-phenyl), 7.71 (8H, m, m-phenyl), 2.83 (12H, s, p-CH_3_), 6.93 (1H, q, 4-phenyl SA), 6.22 (4H, q, 5-phenyl SA), 6.15 (4H, d, 3-phenyl SA), 6.04 (1H, d, 6-phenyl SA).

*(4) 5-SSAZr(p-CH*_3_ *TPP).* Brown red solid. Anal. Calcd. for C_55_H_40_N_4_O_6_SZr(%): C 67.67, H 4.13, N 5.39, S 3.28. Found: C 68.14, H 4.21, N 5.56, S 3.88. UV-vis (CHCl_3_)*λ*_max⁡_: 423 nm (Soret band), 551 nm, 593 nm (Q-band). ESI-MS (CH_3_OH:CH_3_CN): *m/z *calcd for C_55_H_40_N_4_O_6_SZr: 974. Found 975 ([M+H]^+^). IR (KBr)*ν*_max⁡_: 517 cm^−1^ (*ν*_Zr−N_), 685 cm^−1^ (*ν*_Zr−O_, phenolic SA), 715 cm^−1^ (*ν*_Zr−O_, carboxylic 5-SSA). ^1^H NMR (500 MHz, CDCl_3_): *δ*(ppm) 8.96 (8H, s, pyrrole), 8.47 (4H, s, o-phenyl), 8.18 (4H, s, o-phenyl), 7.78 (8H, m, m-phenyl), 2.85 (12H, s, p-CH_3_), 7.06 (1H, q, 4-phenyl 5-SSA), 7.08 (1H, d, 3-phenyl 5-SSA), 7.17 (1H, s, 6-phenyl 5-SSA).

*(5) SAZr(p-ClTPP).* Red solid. Anal. Calcd. for C_51_H_28_Cl_4_N_4_O_3_Zr(%): C 62.64, H 2.89, N 5.73. Found: C 60.12, H 2.84, N 10.97; UV-vis spectra (CHCl_3_)*λ*_max⁡_: 421 nm (Soret band), 551 nm, 594 nm (Q-band). ESI-MS (CH_3_OH:CH_3_CN): *m/z *calcd for C_51_H_28_Cl_4_N_4_O_3_Zr: 978; found 979 ([M+H]^+^). IR (KBr)*ν*_max⁡_: 488 cm^−1^ (*ν*_Zr−N_), 658 cm^−1^ (*ν*_Zr−O_, phenolic SA), 721 cm^−1^ (*ν*_Zr−O_, carboxylic SA). ^1^H NMR (500 MHz, CDCl_3_): *δ* (ppm) 9.12 (8H, s, pyrrole), 8.42 (4H, d, o-phenyl), 8.17 (4H, m, o-phenyl), 8.02 (8H, m, m-phenyl), 6.98 (1H, q, 4-phenyl SA), 6.30 (1H, q, 5-phenyl SA), 6.26 (4H, d, 3-phenyl SA), 6.16 (1H, d, 6-phenyl SA).

*(6) 5-SSAZr(p-ClTPP).* Brown solid. Anal. Calcd. for C_51_H_28_Cl_4_N_4_O_6_SZr(%): C 57.90, H 2.67, N 5.30, S 3.03. Found: C 55.64, H 2.53, N 10.23, S 3.32. UV-vis (CHCl_3_)*λ*_max⁡_: 421 nm (Soret band), 552 nm, 595 nm (Q-band). ESI-MS (CH_3_OH:CH_3_CN): *m/z *calcd. for C_51_H_28_Cl_4_N_4_O_6_SZr: 1057. Found 1058 ([M+H]^+^). IR (KBr)*ν*_max⁡_: 492 cm^−1^ (*ν*_Zr−N_), 660 cm^−1^ (*ν*_Zr−O_, phenolic SA), 722 cm^−1^ (*ν*_Zr−O_, carboxylic SA). ^1^H NMR (500 MHz, CDCl_3_): *δ* (ppm) 9.25 (8H, s, pyrrole), 8.46 (4H, s, o-phenyl), 8.23 (4H, s, o-phenyl), 8.09 (8H, s, m-phenyl), 7.09 (1H, q, 4-phenyl 5-SSA), 7.15 (1H, d, 3-phenyl 5-SSA), 7.20 (1H, s, 6-phenyl 5-SSA).

*(7) SAZr(p-NO*_2_ *TPP).* Red solid. Anal. Calcd. for C_51_H_28_N_8_O_11_Zr(%): C 60.05, H 2.77, N 10.99. Found: C 68.73, H 4.22, N 5.79. UV-vis (CHCl_3_)*λ*_max⁡_: 424 nm (Soret band), 548 nm, 594 nm (Q-band). ESI-MS (CH_3_OH:CH_3_CN): *m/z *calcd. for C_51_H_28_N_8_O_11_Zr: 1020; found 1021 ([M+H]^+^). IR (KBr)*ν*_max⁡_: 483 cm^−1^ (*ν*_Zr−N_), 654 cm^−1^ (*ν*_Zr−O_, phenolic SA), 724 cm^−1^ (*ν*_Zr−O_, carboxylic SA). ^1^H NMR (500 MHz, CDCl_3_): *δ*(ppm) 9.17 (8H, s, pyrrole), 8.42 (4H, d, o-phenyl), 8.07 (4H, d, o-phenyl), 7.64 (8H, s, m-phenyl), 7.05 (1H, q, 4-phenyl SA), 6.55 (1H, q, 5-phenyl SA), 6.50 (1H, d, 3-phenyl SA), 6.18 (1H, d, 6-phenyl SA).

*(8) 5-SSAZr(p-NO*_2_ *TPP).* Red solid. Anal. Calcd. for C_51_H_28_N_8_SO_14_Zr(%): C 55.68, H 2.57, N 10.19, S 2.91. Found: C 63.89, H 3.86, N 5.45, S 3.21. UV-vis (CHCl_3_)*λ*_max⁡_: 425 nm (Soret band), 550 nm, 594 nm (Q-band). ESI-MS (CH_3_OH:CH_3_CN): *m/z *calcd. for C_51_H_28_N_8_SO_14_Zr: 1100; found 1101 ([M+H]^+^). IR (KBr)*ν*_max⁡_: 487 cm^−1^ (*ν*_Zr−N_), 667 cm^−1^ (*ν*_Zr−O_, phenolic SA), 728 cm^−1^ (*ν*_Zr−O_, carboxylic SA). ^1^H NMR (500 MHz, CDCl_3_): *δ*(ppm) 9.26 (8H, s, pyrrole), 8.45 (4H, s, o-phenyl), 8.11 (4H, d, o-phenyl), 7.65 (8H, m, m-phenyl), 7.23 (1H, q, 4-phenyl 5-SSA), 7.78 (1H, d, 3-phenyl 5-SSA), 7.92 (1H, s, 6-phenyl 5-SSA).

## 3. Results and Discussion

### 3.1. Synthesis and Characterization

**Scheme 1:**
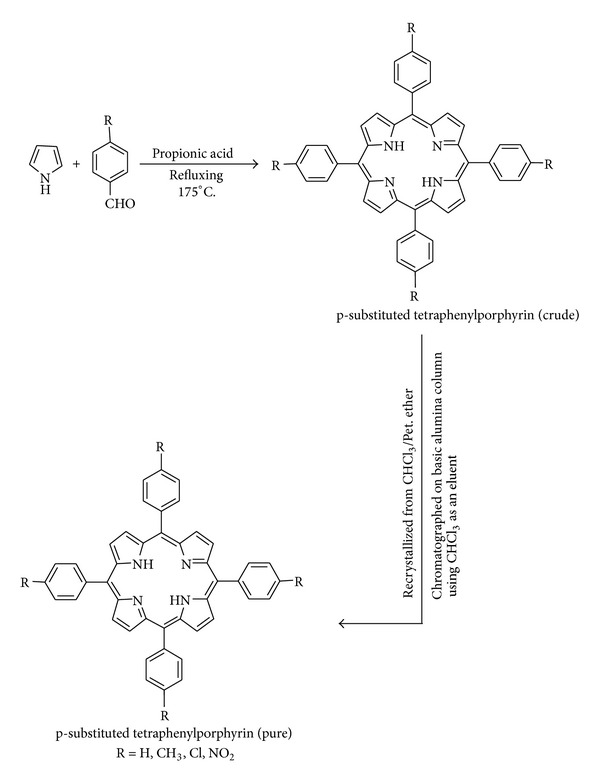
General synthetic route for the synthesis of tetraphenylporphyrin and its parasubstituted derivatives.

**Scheme 2:**
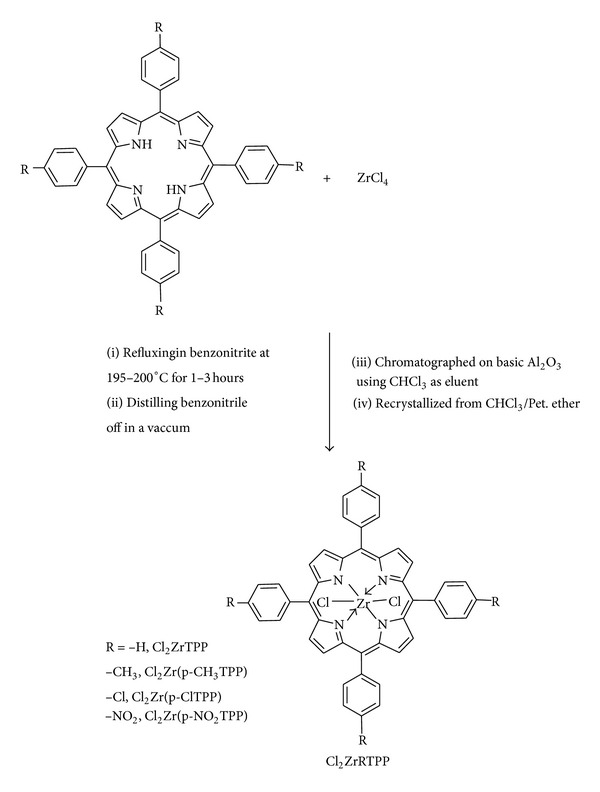
General synthetic route for the synthesis of dichloro(tetraarylporphinato)zirconium(IV).

**Scheme 3:**
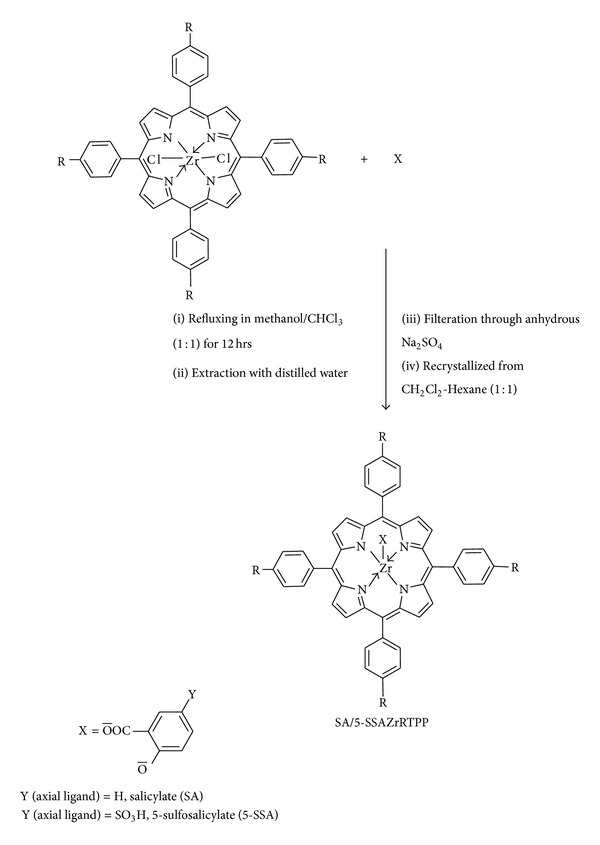
General synthetic route for the synthesis of axially ligated Zr(IV) porphyrins complexes.

The general synthetic routes to peripherally tetrasubstituted tetraphenylporphyrin derivatives (RTPP), their corresponding metallated, and axially ligated zirconium(IV) porphyrins are shown in Schemes 1, 2, and 3, respectively. All of these new zirconium(IV) porphyrins were purified by column chromatography with aluminum oxide as adsorbent and were characterized by spectral data (UV-visible spectroscopy, IR spectroscopy, ^1^H NMR spectroscopy, mass spectral data, and elemental analysis). The characterization data of the new compounds are consistent with the assigned formula. All the free base porphyrins, substituted tetraphenylporphyrin zirconium(IV) chlorides, and substituted tetraphenylporphyrin zirconium(IV) salicylates, are water insoluble.

#### 3.1.1. Spectral Analysis of Cl_2_Zr(IV)RTPP and SA/5-SSAZr(IV)RTPP

The structures of all free base porphyrins (RTPP), substituted tetraphenylporphyrin zirconium(IV) chlorides Cl_2_Zr(IV)RTPP and substituted tetraphenylporphyrin zirconium(IV) salicylates (SA/5-SSAZr(IV)RTPP), are characterized by UV-vis, and the spectral data are listed in Tables 1 and 2. The UV-vis data shows that many of the absorption bands of the parasubstituted derivatives exhibit small shifts to longer wavelength, that is, bathochromic shift (red shift) as compared to the spectrum of TPP. The most pronounced bathochromic shift occurs in p-NO_2_TPP with paranitro group on mesophenyl ring of the porphyrin but in all the substituted tetraphenylporphyrin, intensities of all the peaks are higher than those of the parent tetraphenylporphyrin. The reason might be that the strong electron-withdrawing-NO_2_ group decreased the electronic density of the porphyrin ring. Thus, the *π*-π* electron excitation of the porphyrin ring required absorbing the light of smaller energy (longer wavelength).

**Figure 1:**
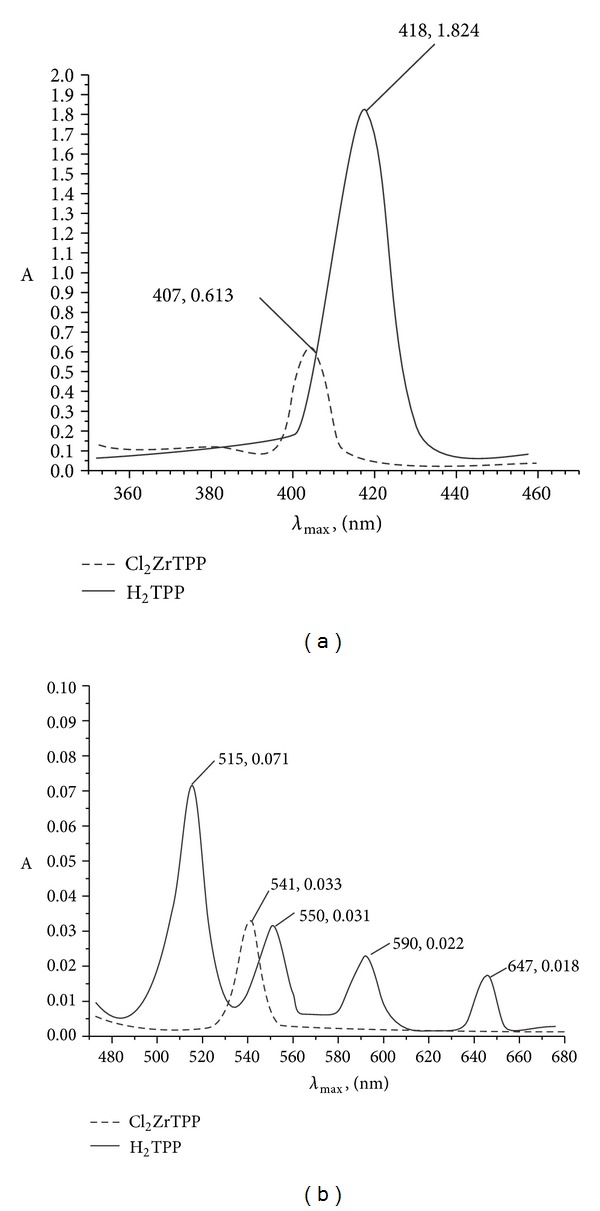
UV-Vis overlapped (a) B bands (b) Q bands of TPP and (Cl)_2_ZrTPP in chloroform.

**Figure 2:**
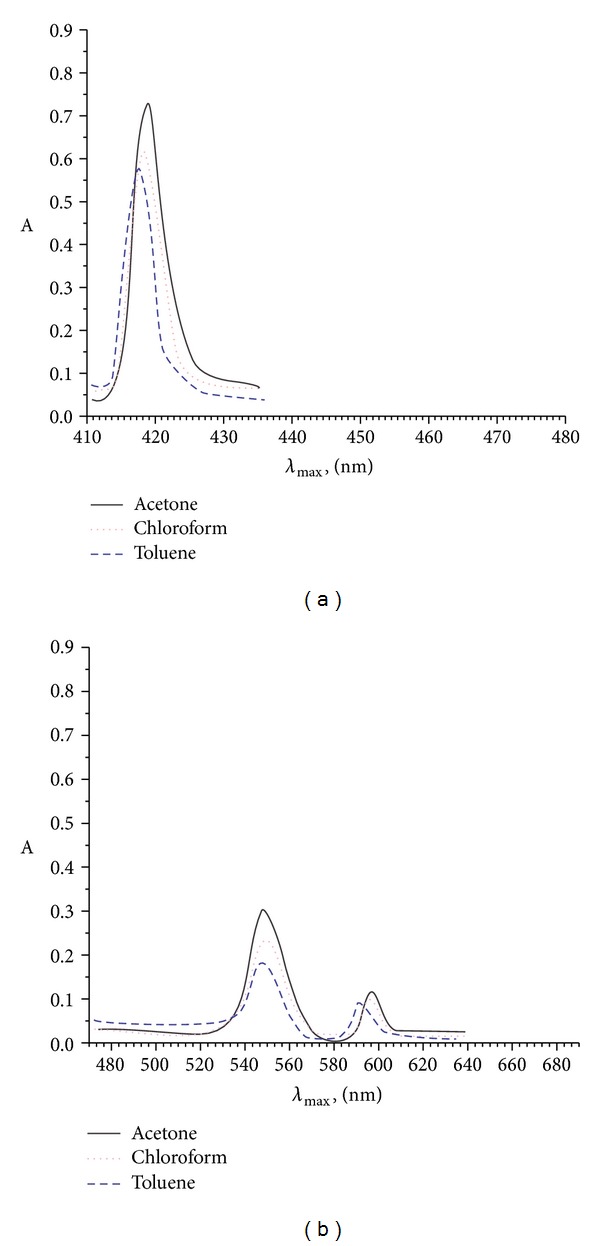
UV-vis spectra of SAZrTPP (a) B bands (b) Q bands in different solvents.

By comparing the UV-vis data in Table 1, it was noticed that in the visible absorption spectra, free base porphyrins show an intense absorption, referred to as the Soret band at around 400 nm and four weaker absorptions known as Q bands between 450 nm and 800 nm. When the metal ion was inserted into the porphyrin ring, the number and intensity of the Q bands were found to decrease and the B (Soret) band showed a slight blue shift (Figure 1). The reason might be that the structural symmetry of zirconium(IV) porphyrin compounds was improved and the energy gap decreased as compared to the free base porphyrins [29]. In axially ligated zirconium(IV) porphyrin complexes, both B- and Q-bands regions of the spectra show slight red shift. The red shift may be due to structural distortion in the porphyrin macrocycle and concomitant electronic coupling of the metalloporphyrin to the salicylate mediated by the zirconium metal ion [18]. The optical absorption spectra of the compounds of the axially ligated zirconium(IV) porphyrins were when recorded in different solvents; only marginal changes of *λ*_max⁡_ values, absorption coefficient (*ε*), and oscillatory strength (*f*) were observed. It is found that with the increase in polarity of the solvents, B- and Q-bands in axially ligated Zr(IV) metal derivatives with different porphyrins show a slight red shift with progressive broadening of bands indicating that the magnitude of red shift of B- and Q-bands depends on the nature of the solvent used. This is so since excited state is more polar than the ground state and hence stabilization is greater relative to the ground state in polar solvents. It is observed that for all the axially ligated Zr(IV) derivatives, B- and Q-bands exhibit a red shift on increasing the polarity of the solvents in the order: acetone > chloroform > toluene (Figure 2). The magnitude of change of the “*f*” values in axially ligated Zr(IV) metal derivatives of porphyrins reveal the relative strength of interaction.

Infrared spectral data of above porphyrin compounds is listed in Table 3. It is found that the *ν*(N–H) stretching and bending frequencies of free base porphyrins are located at ~3400–3320 cm^−1^ and ~960 cm^−1^, respectively [30]. When the zirconium ion was inserted into the porphyrin ring, the N–H vibration frequency of free base porphyrins disappeared and the characteristic *ν*(Zr–N) vibration frequency was found at ~500–400 cm^−1^, which indicated the formation of zirconium(IV) porphyrin compounds [31]. In the spectra of all the axially ligated zirconium(IV) porphyrin complexes, the band due to *ν*(O–H) of the ligand disappeared indicating the coordination of phenolic oxygen to the metal via deprotonation [32]. The band assigned to the *ν*(C–O) stretching show upward shift in the range ~1264–1249 cm^−1^, which is due to the coordination of the phenolic oxygen to Zr(IV) porphyrins [33]. The incorporation of salicylates in Zr(IV) metal derivatives of different porphyrins, that is, SA/5-SSAZrRTPP, is further confirmed by the appearance of Zr–O vibrational frequencies in the range 690–662 cm^−1^ and 740–719 cm^−1^ corresponding to the ligation of zirconium to oxygen of phenolic and carboxylic groups of salicylate, respectively. Thus, the zirconium atom in the centre of porphyrin ring coordinates with the salicylate group axially to form six-coordinate complex of Zr(IV) porphyrin.

^1^H NMR data of free base porphyrin, their corresponding metallated, and axially ligated zirconium(IV) porphyrin complexes in CDCl_3_ at 298 K is listed Table 4. In all the metallated porphyrins, there was absence of signal related to N–H protons and shift in other signals indicating the insertion of zirconium in porphyrin macrocycle [31]. Generally, the presence of Zr(IV) metal in the porphyrin ring shifts the resonances of the porphyrin's protons to downfield accompanied by marginal changes in the pattern. One of the important features of axially ligated Zr(IV) derivatives of porphyrins is that the metal is almost out of the plane of the porphyrin ring which has earlier been reviewed in the literature, responsible for the production of asymmetric environment above and below the plane of the macrocycle which ultimately accounts for the pronounced nonequivalence of the orthoprotons of the phenyl rings. In axially ligated zirconium(IV) porphyrin complexes, the signals of axial salicylic and 5-sulfosalicylic fragment protons are shifted to higher field in comparison to the signals of porphyrin protons and also in comparison to proton signals of free salicylic and 5-sulfosalicylic acids respectively [34]. These positions of protons show that axial ligand is under the influence of *π*-conjugated system of porphyrin macrocycle [22]. This is most probably due to deshielding effect resulting from the **σ**-donation of electron density upon bond formation as compared to the shielding effect of the porphyrin.

Mass spectrometric characterization of SA/5-SSAZrRTPP complexes employed ESI as soft ionization technique. The mass spectra of axial ligated zirconium(IV) porphyrins with salicylic acid/5-sulfosalicylic acid are characterized by the presence of the molecular ion peak for monomeric form followed by a degree of fragmentation when employing this technique, which suggested that axial ligand was labile. The substituents such as sulphonates in the axial ligand were labile too. The major losses are 81, 104, and 33 corresponding to the loss of SO_3_H group, salicylic fragment, and H_2_O molecules. The obtained ESI-MS data display that the molecule of axially ligated zirconium(IV) porphyrins contain one or two molecules of solvent coordinated to the central zirconium atom, which are not labile sufficiently and present in all molecular ions. This fact can be explained by the coordinated unsaturation of zirconium atom [22].

**Figure 3:**
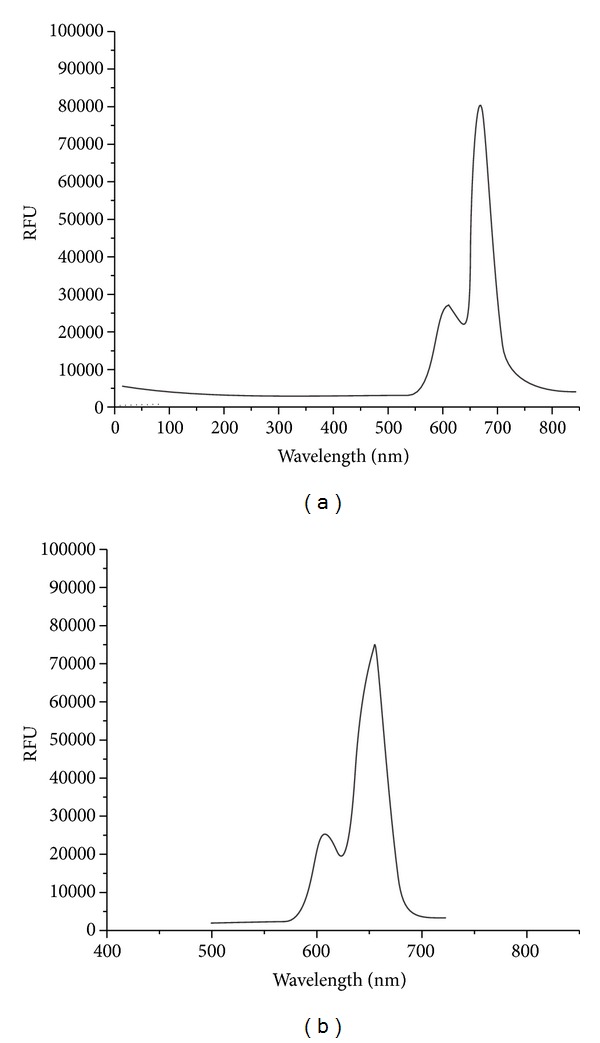
S_1_ → S_0_ fluorescence spectrum of (a) SAZr(p-CH_3_TPP) and (b) 5-SSAZr(p-CH_3_TPP) in DMSO (C = 10^−6^ mol L^−1^, *λ*_exc_ = 420 nm).

In the present investigation, the variations of emission properties in free base porphyrin p-CH_3_TPP and its corresponding metallated and axially ligated Zr(IV) porphyrins have been studied (Table 5). The freebase porphyrins exhibit two emission bands at 653 and 715 nm corresponding to Q(0,0) and Q(0,1) transitions, respectively, the intensity of the Q(0,0) being higher than the Q(0,1) transition. The complexes Cl_2_Zr(p-CH_3_TPP), SAZr(p-CH_3_TPP), and 5-SSAZr(p-CH_3_TPP) are emissive and show intraligand fluorescence comparable to other regular metalloporphyrins with maxima occurring at 653, 660, and 657 nm, respectively. However, the emission bands of axially ligated Zr(IV) porphyrins are blue shifted compared to free base porphyrins. This behavior is attributed to an enhanced spin-orbit coupling induced by the presence of the heavy-atom central metals in zirconium(IV) porphyrins complexes, which leads to a more efficient intersystem crossing from the lowest porphyrin singlet excited state ^1^S_1_(*π*, π*) to the corresponding triplet manifold and thus reduces the probability of fluorescent emission [35]. Thus, the excitation spectrum of fluorescence is in agreement with absorption spectrum (Figure 3).

**Figure 4:**
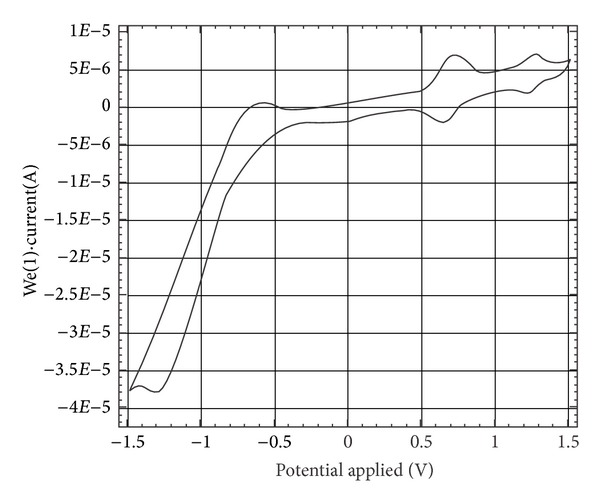
Cyclic voltammogram of Zr(p-CH_3_TPP) in CH_2_Cl_2_ containing 0.1 M (TBA)PF_6_. Scan rate 20 mV/s.

Cyclic voltammetric studies have been carried out for Cl_2_Zr(p-CH_3_TPP) and its salicylate and 5-sulfosalicylate ligated Zr(IV) derivatives to elucidate the effects of the peripheral substituents on the electrochemical redox potentials (Table 6). The electrochemical redox processes of the porphyrin and their metallated derivatives arise primarily from the *π* electron system of the macrocycle.  Figure 4 shows the cyclic voltammogram of Cl_2_Zr(p-CH_3_TPP) in CH_2_Cl_2_. In the oxidative scan, two redox couples were observed at 0.66 and 1.22 V corresponding to the formation of porphyrin cation radical and porphyrin dication, respectively. There is a wave in the reductive scan at 1.30 V. The wave is characteristic of one electron reduction of the macrocycle. The data clearly demonstrated the formation of the porphyrin radical anion and inability to reduce Zr(IV) metal centre [36].

For the investigated salicylate ligated zirconium(IV) porphyrins, we observed one reversible reduction wave and two reversible oxidation waves. The three waves for the complexes (Table 6) can be attributed to one-electron processes ZrT(p-CH_3_)PPX^0/−1^, ZrT(p-CH_3_)PPX^1+/0^, and ZrT(p-CH_3_)PPX^2+/+1^ (X = SA, 5-SA), respectively, corresponding to the one-electron oxidation/reduction processes involving not only the porphyrin ring but also the entire system as a whole, including even the conjugated salicylate/5-sulfosalicylate ligand [37].

The central metal atom (zirconium), being electrochemically passive [38], plays the role of a “linkage” connecting the-conjugated systems into a whole integral. The zirconium(IV) porphyrin HOMO-LUMO gap can be expressed as Δ*E*, the potential difference between the first oxidation and the first reduction. The differences between the potentials of the first oxidation wave and the first reduction wave (*E*_ox1_ − *E*_red1_) are 1.93 V and 1.98 V for SAZr(p-CH_3_TPP) and 5-SSAZr(p-CH_3_TPP), respectively, allow us to assign these waves to oxidation/reduction processes involving the macrocyclic ring. The experimental Δ*E* values of substituted zirconium(IV) porphyrins are in the range of 1.88–1.98 V.

**Figure 5:**
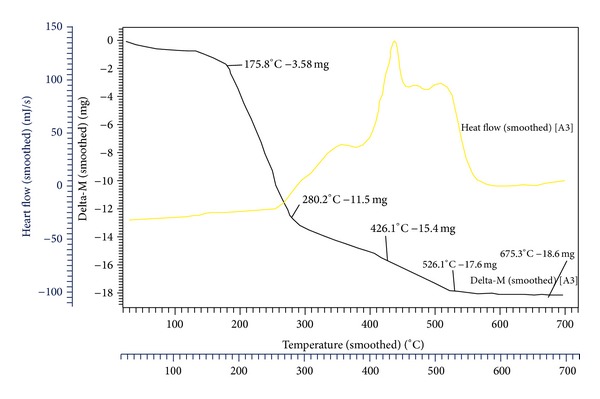
TGA (black line) and DTA (yellow line) picture of SAZrTPP.

Thermal analysis of SAZrTPP is shown in Figure 5. The TG curve of SAZrTPP (Figure 5) shows a continuous weight loss starting from 150°C to 700°C, when a stable oxide of ZrO_2_ is formed. The initial weight loss of about 16.37% observed between 140°C and 175.8°C is attributed to the loss of salicylate group (the theoretical value = 16.32%). In the range of 200 to 280.2°C, up to 52.58% of the mass had been lost due to the loss of tetraphenyl groups (the theoretical value = 53.04%). At 426.1°C, up to 70.41% (the theoretical value = 69.01%) of the total mass had been lost which is attributed to the collapse of macrocyclic ligand; correspondingly, there is a large exothermic peak in DTA curve. The organic moiety decomposes further with increasing temperature. Further in the range 425–526.2°C, the weight loss reaches up to 80.47% which is attributed to the removal of pyrrole fragments. At 675.3°C the evident weight loss of 85.04% is due to the complete decomposition of SAZrTPP leaving behind stable zirconium oxide (the theoretical value = 85.33%). Simultaneously, there are some exothermal peaks in the range of 340–530°C on the DTA curve showing major weight loss in the region. The small exothermic peaks correspond to the loss of chains of the porphyrin ring and the large exothermic peak corresponds to the collapse of the porphyrin skeleton. The thermogravimetric analysis of the complex showed that zirconium(IV) porphyrin complex is stable up to approximately 150°C.

#### 3.1.2. Biological Studies

The ability of porphyrins to accumulate in malignant tumors has led to the application of these compounds in diagnosis and treatment of these malignant neoplasms. In the present research work, some complexes were evaluated for antibacterial and anticancer activity. Antibacterial activity of the synthesized zirconium(IV) porphyrin complexes was tested by Agar well-diffusion method (Table 7). The free base porphyrin and their corresponding metallated and axially ligated zirconium(IV) porphyrin complexes, namely, TPP, p-CH_3_TPP, p-NO_2_TPP, Cl_2_ZrTPP, Cl_2_Zr(p-CH_3_TPP), Cl_2_Zr(p-NO_2_TPP), SAZrTPP, 5-SSAZrTPP, SAZr(p-CH_3_TPP), 5-SSAZr(p-CH_3_TPP), SAZr(p-NO_2_TPP), and 5-SSAZr(p-NO_2_TPP) were tested at the concentration of 100 *μ*g/mL against five bacterial strains, namely, *Bacillus subtilis, Micrococcus luteus, Staphylococcus aureus, Pseudomonas fluorescens, *and* Escherichia coli.* Our results demonstrated antibacterial activity against most of the zirconium(IV) porphyrin complexes. Among all the complexes prepared, 5-SSAZr(p-NO_2_TPP) was found to be highly potential against all the five bacterial strains with sensitivity ranging from 10 to 15 mm zone of inhibition at 100 *μ*g/mL, respectively (Table 7). SAZr(p-CH_3_TPP) complex showed sensitivity only against *B. subtilis *and *M. luteus* with zones of inhibition of 6 and 7 mm, respectively, and it was the only other complex after 5-SSAZr(p-NO_2_TPP) complex that showed antibacterial sensitivity against gram positive bacteria (*B. subtilis*). Other complexes, namely, Cl_2_ZrTPP, SAZrTPP, p-CH_3_TPP, Cl_2_Zr(p-CH_3_TPP), and 5-SSAZr(p-CH_3_TPP) showed sensitivity only against gram negative bacteria *E. coli* with zones of inhibition ranging from 6 to 14 mm, respectively. The rest of the complexes did not show any sensitivity against any bacterial strain used in the studies (Table 7).

#### 3.1.3. *In Vitro* Cytotoxicity

**Figure 6:**
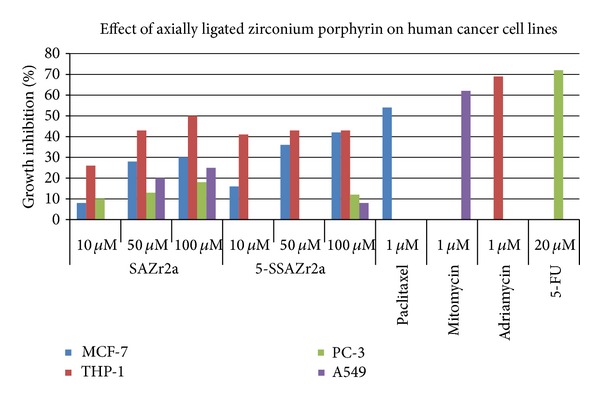
Cytotoxicity of SAZr(p-CH_3_TPP) and 5-SSAZr(p-CH_3_TPP), where 2a = p-CH_3_TPP.

The cytotoxicity of SAZr(p-CH_3_TPP) and 5-SSAZr(p-CH_3_TPP) was evaluated against four human cancer cell lines, namely, breast (MCF-7), leukemia (THP-1), prostate (PC-3), and lung (A549) at different concentrations as shown in Figure 6. It was observed that both compounds showed less than 50% growth inhibition.

## 4. Conclusion

**Figure 7:**
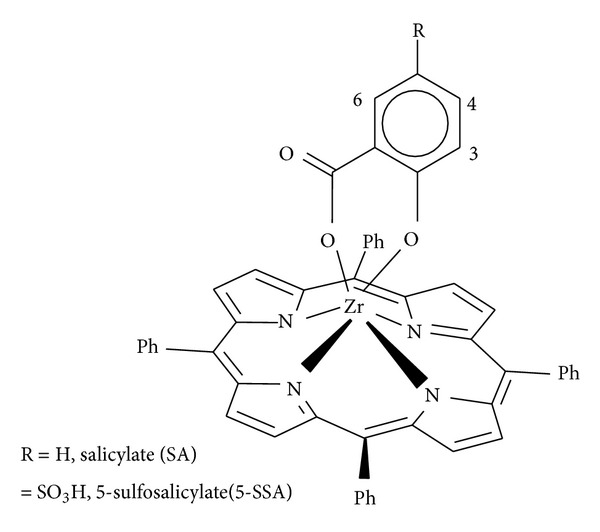
Proposed structure for axially ligated zirconium(IV) porphyrin complexes.

In this paper, we have described the synthesis of pure porphyrins and their subsequent reactions with ZrCl_4_ and salicylic acid/5-sulfosalicylic acid so as to get their axially ligated Zr(IV) porphyrins. The structures of above porphyrin compounds were characterized by UV-vis, IR, ^1^H NMR, and elemental analysis. In axially ligated zirconium(IV) porphyrin complexes, bands showed slight red shift corresponding to the structural distortion in the porphyrin macrocycle and concomitant electronic coupling of the metalloporphyrin to the salicylate mediated by the zirconium metal ion. The infrared spectra of these compounds showed that salicylate groups axially ligated to zirconium(IV) porphyrins to form six-coordinate complexes of Zr(IV) porphyrins (Figure 7). Additionally, the ^1^H NMR spectral study of these compounds showed that signals of axial salicylic and 5-sulfosalicylic fragment protons are shifted to higher field in comparison to the signals of porphyrin protons and also in comparison to proton signals of free salicylic and/or 5-sulfosalicylic acids respectively. The ESI mass spectroscopy provided the information regarding the appearance of the molecular ion peak (*m*/*z*) and specific fragmentation. Meanwhile, cyclic voltammograms of axially ligated zirconium(IV) porphyrins complexes have shown three waves for the studied complexes which can be attributed to one-electron processes M(4-CH_3_TPP)X^0/−1^, M(4-CH_3_TPP)X^1+/0^, and M(4-CH_3_TPP)X^2+/+1^(X = SA, 5-SA; M = Zr). All these complexes exhibit reversible oxidation and reduction potentials characteristic of the porphyrin. The data of thermogravimetric analysis indicated that the zirconium(IV) porphyrins complexes are stable up to approximately 150°C. Their possible biological activities were also investigated and most of the samples exhibited moderate antimicrobial activity against test microorganisms. The two complexes screening for anticancer activity showed less than 50% growth inhibition.

## Figures and Tables

**Table 1:** UV-vis data of free base porphyrins and corresponding tetraphenylporphyrin zirconium(IV) chlorides complexes in chloroform.

No.	Compound	*λ*_max⁡_ (nm)	
		B-band	Q-band
1	TPP	418	515, 552, 590, 646
2	p-CH_3_TPP	419	517, 552, 592, 646
3	p-ClTPP	422	519, 555, 590, 655
4	p-NO_2_TPP	423	516, 552, 592, 648
5	Cl_2_ZrTPP	407	541
6	Cl_2_Zr(p-CH_3_TPP)	409	540
7	Cl_2_Zr(p-ClPP)	416	540
8	Cl_2_Zr(p-NO_2_TPP)	416	542

**Table 2:** UV-vis data of salicylate/5-sulfosalicylate ligated zirconium(IV) complexes in different solvents.

Complex	Solvent	*λ*_max⁡_, nm (log *ε*, M^−1^ cm^−1^)			*ν*_1/2_ (cm^−1^)		*f*
		B (0, 0)	Q (1, 0)	Q (0, 0)	B (0, 0)	Q (1, 0)	B (0, 0)
SAZrTPP	Acetone	420 (4.8691)	549 (3.544)	586 (2.278)	1123	792.3	0.351
	Chloroform	418 (4.7862)	549 (3.362)	590 (2.398)	1134	796.6	0.307
	Toluene	417 (4.7686)	547 (3.255)	585 (2.176)	1151	808.4	0.292

5-SSAZrTPP	Acetone	422 (4.8651)	550 (3.579)	590 (2.954)	1120	796.6	0.352
	Chloroform	420 (4.8463)	549 (3.414)	587 (2.903)	1123	794.9	0.340
	Toluene	417 (4.8331)	545 (3.301)	585 (2.778)	1135	802.5	0.335

SAZr(p-CH_3_TPP)	Acetone	424 (4.8847)	548 (3.362)	593 (2.994)	1110	799.6	0.368
	Chloroform	422 (4.8691)	547 (3.278)	591 (2.972)	1123	800.8	0.350
	Toluene	419 (4.8475)	547 (3.230)	589 (2.968)	1138	802.2	0.347

5-SSAZr(p-CH_3_TPP)	Acetone	425 (4.8948)	553 (3.672)	594 (3.064)	1106	785.2	0.370
	Chloroform	423 (4.8615)	551 (3.362)	593 (3.334)	1118	789.3	0.353
	Toluene	418 (4.8518)	547 (3.447)	590 (3.230)	1143	802.5	0.351

SAZr(p-ClTPP)	Acetone	423 (4.8651)	553 (3.518)	596 (2.976)	1151	785.1	0.365
	Chloroform	421 (4.8312)	551 (3.322)	594 (2.970)	1127	788.3	0.331
	Toluene	419 (4.7951)	551 (3.114)	590 (2.930)	1134	790.7	0.304

5-SSAZr(p-ClTPP)	Acetone	424 (4.8759)	552 (3.518)	597 (3.079)	1103	787.7	0.357
	Chloroform	421 (4.8318)	552 (3.342)	595 (2.973)	1126	793.8	0.331
	Toluene	421 (4.7951)	549 (3.176)	590 (2.942)	1129	796.7	0.305

SAZr(p-NO_2_TPP)	Acetone	424 (4.9647)	551 (3.5051)	596 (3.041)	1110	790.0	0.328
	Chloroform	424 (4.7331)	548 (3.397)	594 (2.984)	1125	798.7	0.292
	Toluene	422 (4.6599)	547 (3.230)	593 (2.943)	1129	799.6	0.291

5-SSAZr(p-NO_2_TPP)	Acetone	423 (4.9498)	547 (3.447)	598 (3.149)	1106	787.6	0.330
	Chloroform	425 (4.7259)	550 (3.362)	594 (3.124)	1120	793.3	0.306
	Toluene	422 (4.6541)	547 (3.204)	594 (3.082)	1128	802.1	0.267

**Table 3:** Main infrared absorption frequencies of free base porphyrins, their corresponding metallated, and axially ligated Zr(IV) porphyrin complexes.

Compound	IR (cm^−1^)										
	*ν*_N–H_	*ν*_C–H_	*ν*_Zr–O_	*γ*_C–H_	*ν*_C=N_	*ν*_C=O_	*ν*_Zr–N_	*ν*_SO_2__	*ν*_PhCO_	*ν*_Zr–O_	*ν*_OCO_
TPP	3326	2963	—	797	1349	—	—	—	—	—	—
Cl_2_ZrTPP	—	2960	—	800	1351	—	485	—	—	—	—
SAZrTPP	—	2961	662	800	1328	1731	485	—	1260	719	1379
5-SSAZrTPP	—	2960	678	801	1336	1730	500	1156, 1074	1264	722	1378
p-CH_3_TPP	3416	2964	—	794	1350	—	—	—	—	—	—
Cl_2_Zr(p-CH_3_TPP)	—	2955	—	800	1339	—	508	—	—	—	—
SAZr(p-CH_3_TPP)	—	2956	683	800	1335	1735	510	—	1260	713	1377
5-SSAZr(p-CH_3_TPP)	—	2954	685	803	1329	1729	517	1172, 1119	1261	715	1379
p-ClTPP	3428	2964	—	797	1354	—	—	—	—	—	—
Cl_2_Zr(p-ClTPP)	—	2962	—	803	1338	—	485	—	—	—	—
SAZr(p-ClTPP)	—	2961	658	801	1328	1729	488	—	1258	721	1377
5-SSAZr(p-ClTPP)	—	2961	660	802	1336	1726	492	1181, 1124	1260	722	1378
p-NO_2_TPP	3449	2964	—	800	1329	—	—	—	—	—	—
Cl_2_Zr(p-NO_2_TPP)	—	2952	—	802	1332	—	482	—	—	—	—
SAZr(p-NO_2_TPP)	—	2945	654	800	1335	1728	483	—	1259	724	1375
5-SSAZr(p-NO_2_TPP)	—	2938	667	806	1337	1725	487	1174, 1124	1261	728	1378

**Table 4:** ^
1^H NMR data of free base porphyrin and their zirconium(IV) porphyrins complexes in CDCl_3 _at 298 K.

Compound	Porphyrin's *δ*, ppm			Salicylate protons *δ*, ppm
	N–H	*β*-H	Meso-H	
TPP	−2.76 (s, 2H)	8.82 (s, 8H)	8.27 (s, 8H, H_o_) 7.60 (s, 12H, H_m,p_)	—

Cl_2_ZrTPP		8.87 (s, 8H)	8.32 (d, 4H, H_o_) 8.04 (d, 4H, H_o_) 7.64 (s, 12H, H_m,p_)	—

SAZrTPP		8.93 (s, 8H)	8.50 (s, 4H, H_o_) 8.07 (d, 4H, H_o_) 7.68 (m, 12H, H_m,p_)	6.06 (d, H_6_) 6.18 (q, H_5_) 6.98 (q, H_4_) 6.16 (m, H_3_)

5-SSAZrTPP		8.96 (s, 8H)	8.53 (s, 4H, H_o_) 8.11 (s, 4H, H_o_) 7.71 (m, 12H, H_m,p_)	7.19 (s, 1H, H_6_) 7.15 (d, 1H, H_3_) 7.10 (q, 1H, H_4_)

p-CH_3_TPP	−2.75 (s, 2H)	8.78 (s, 8H)	8.18 (s, 8H, H_o_) 7.56 (s, 8H, H_m_) 2.64 (s, 12H, H_CH3_)	—

Cl_2_Zr(p-CH_3_TPP)		8.80 (s, 8H)	8.28 (d, 4H, H_o_) 8.00 (s, 4H, H_o_) 7.65 (s, 8H, H_m_) 2.70 (s, 12H, H_CH3_)	—

SAZr(p-CH_3_TPP)		8.94 (s, 8H)	8.42 (d, 4H, H_o_) 8.15 (m, 4H, H_o_) 7.71 (m, 8H, H_m_) 2.83 (s, 12H, H_CH_3__)	6.04 (d, H_6_) 6.22 (q, H_5_) 6.93 (q, H_4_) 6.15 (d, H_3_)

5-SSAZr(p-CH_3_TPP)		8.96 (s, 8H)	8.47 (s, 4H, H_o_) 8.18 (s, 4H, H_o_) 7.78 (m, 8H, H_m_) 2.85 (s, 12H, H_CH_3__)	7.17 (s, 1H, H_6_) 7.08 (d, 1H, H_3_) 7.06 (q, 1H, H_4_)

p-ClTPP	−2.75 (s, 2H)	8.73 (s, 8H)	8.25 (s, 4H, H_o_) 7.75 (s, 4H, H_p_)	—

Cl_2_Zr(p-ClTPP)		8.97 (s, 8H)	8.33 (d, 4H, H_o_) 8.13 (s, 4H, H_o_) 7.59 (s, 8H, H_m_)	—

SAZr(p-ClTPP)		9.12 (s, 8H)	8.42 (d, 4H, H_o_) 8.17 (m, 4H, H_o_) 8.02 (m, 8H, H_m_)	6.16 (d, H_6_) 6.30 (q, H_5_) 6.98 (q, H_4_) 6.26 (d, H_3_)

5-SSAZr(p-ClTPP)		9.25 (s, 8H)	8.46 (s, 4H, H_o_) 8.23 (s, 4H, H_o_) 8.09 (s, 8H, H_m_)	7.20 (s, 1H, H_6_) 7.15 (d, 1H, H_3_) 7.09 (q, 1H, H_4_)

p-NO_2_TPP	−2.84 (s, 2H)	8.83 (s, 8H)	8.68 (s, 8H, H_o_) 8.41 (s, 8H, H_m_)	—

Cl_2_Zr(p-NO_2_TPP)		9.05 (s, 8H)	8.42 (d, 4H, H_o_) 8.04 (d, 4H, H_o_) 7.79 (s, 8H, H_m_)	—

SAZr(p-NO_2_TPP)		9.17 (s, 8H)	8.42 (s, 4H, H_o_) 8.07 (d, 4H, H_o_) 7.64 (s, 8H, H_m_)	6.18 (d, H_6_) 6.55 (q, H_5_) 7.05 (q, H_4_) 6.52 (d, H_3_)

5-SSAZr(p-NO_2_TPP)		9.26 (s, 8H)	8.45 (s, 4H, H_o_) 8.11 (s, 4H, H_o_) 7.65 (s, 8H, H_m_)	7.92 (s, 1H, H_6_) 7.78 (d, 1H, H_3_) 7.23 (q, 1H, H_4_)

**Table 5:** Summary of the fluorescence band maxima at 23 K in DMSO.

Compound	*λ*_max⁡_, nm		
	B (0, 0)	Q (0, 0)	Q (0, 1)
p-CH_3_TPP	450	653	715
Cl_2_Zr(p-CH_3_TPP)	441	—	653
SAZr(p-CH_3_TPP)	440	609	660
5-SSAZr(p-CH_3_TPP)	440	608	657

**Table 6:** Electrochemical data for synthesized Cl_2_Zr(p-CH_3_TPP), SAZr(p-CH_3_TPP), and 5-SSAZr(p-CH_3_TPP) complexes in dichloromethane.

Species	*E*_OX2_/V	*E*_OX1_/V	*E*_red1_/V	Δ*E* d/V
Cl_2_Zr(4-CH_3_TPP)	1.22	0.66	1.30	1.88
SAZr(4-CH_3_TPP)	1.25	0.68	1.22	1.93
5-SSAZr(4-CH_3_TPP)	1.28	0.70	1.15	1.98

**Table 7:** *In vitro* antibacterial evaluation of free base porphyrin and the corresponding zirconium(IV) porphyrin complexes.

Porphyrin (100 *µ*g/mL)	*B. subtilis *	*S. aureus *	*M. luteus *	*E. coli *	*P. fluorescens *
TPP	—	—	—	—	—
Cl_2_ZrTPP	—	—	—	7	—
SAZrTPP	—	—	—	14	—
5-SSAZrTPP	—	—	—	—	—
p-CH_3_TPP	—	—	—	6	—
Cl_2_Zr(p-CH_3_TPP)	—	—	—	11	—
SAZr(p-CH_3_TPP)	6	—	7	—	—
5-SSAZr(p-CH_3_TPP)	—	—	—	9	—
(p-NO_2_TPP)	—	—	—	—	—
Cl_2_Zr(p-NO_2_TPP)	—	—	—	—	—
SAZr(p-NO_2_TPP)	—	—	—	—	—
5-SSAZr(p-NO_2_TPP)	10	11	15	13	11

Control chloramphenicol (10 *µ*g)	16	12	18	14	9
